# The medaka mutation *tintachina* sheds light on the evolution of V-ATPase B subunits in vertebrates

**DOI:** 10.1038/srep03217

**Published:** 2013-11-14

**Authors:** Claudia Müller, Ignacio Maeso, Joachim Wittbrodt, Juan R. Martínez-Morales

**Affiliations:** 1 Centro Andaluz de Biología del Desarrollo (CSIC/UPO/JA), 41013 Sevilla, Spain; 2 Centre for Organismal Studies, COS, University of Heidelberg, Heidelberg, Germany; 3 Department of Zoology, University of Oxford, OX1 3PS, United Kingdom; 4 These authors contributed equally to this work.

## Abstract

Vacuolar-type H^+^ ATPases (V-ATPases) are multimeric protein complexes that play a universal role in the acidification of intracellular compartments in eukaryotic cells. We have isolated the recessive medaka mutation *tintachina* (*tch*), which carries an inactivating modification of the conserved glycine residue (*G75R*) of the proton pump subunit *atp6v1Ba/vatB1*. Mutant embryos show penetrant pigmentation defects, massive brain apoptosis and lethality before hatching. Strikingly, an equivalent mutation in *atp6v1B1* (*G78R*) has been reported in a family of patients suffering from distal renal tubular acidosis (dRTA), a hereditary disease that causes metabolic acidosis due to impaired kidney function. This poses the question as to how molecularly identical mutations result in markedly different phenotypes in two vertebrate species. Our work offers an explanation for this phenomenon. We propose that, after successive rounds of whole-genome duplication, the emergence of paralogous copies allowed the divergence of the *atp6v1B* cis-regulatory control in different vertebrate groups.

V-ATPases are highly conserved ATP-dependent proton pumps that play a universal role as pH regulators in intracellular acidic organelles of eukaryotic cells. They form multi-subunit complexes assembled in two subdomains: the V_o_ membrane domain, which is responsible for proton transport across membranes and is comprised of the subunits a, d, c, c″, and e; and the V1 cytoplasmic domain, which is responsible for ATP hydrolysis and is made up of 8 subunit types, designated as A to H. Intracellular V-ATPases play essential roles in receptor-mediated endocytosis, vesicular trafficking between organelles, membrane fusion, protein degradation and autophagy^1^,^2^,^3^. In addition to their role in intracellular compartments, V-ATPases can also pump protons across the plasma membrane, thus acidifying the extracellular medium. This membrane localization has been described in various mammalian cell types including macrophages, osteoclasts and renal intercalated cells. The targeting of V-ATPases to the cell surface is largely mediated by tissue specific a-subunit isoforms. These include a1, a3 and a4, which have been reported at the plasma membrane in neurons, osteoclasts and renal intercalated cells respectively^4^. In addition, the B subunit kidney-specific isoform (*atp6v1B1*) has been shown to interact with the PDZ protein NHERF1 through its C-terminal motif^5^, which in turn regulates the docking of the V-ATPase complex to the apical membrane in renal cells. Interestingly, hereditary diseases linked to mutations in V-ATPase subunits have been reported specifically for these targeting isoforms. Mutations in the osteoclast-specific a3 isoform cause autosomal recessive osteopetrosis^6^, whereas mutations in both the renal-specific a4 and B1 isoforms cause distal renal tubular acidosis^7^,^8^. Similarly, interference with normal proton secretion in the kidney has been observed in *atp6v1B1−/−*mice^9^.

Within the vacuolar proton pump, B subunits are essential components of the central A_3_B_3_ hexameric barrel of the V1 domain, which is directly responsible for ATP hydrolysis^10^. While a single *atp6v1B* gene can be identified in invertebrate genomes^11^, two different paralogous genes have been reported in mammals: *atp6v1B1*, which is restricted to a number of tissues including kidney, lung, inner ear, olfactory epithelium and epididymal cells, and *atp6v1B2*, which is broadly expressed, if not practically ubiquitous^7^,^12^,^13^,^14^. Two different B genes, *vatB1* and *vatB2*, also named *atp6v1Ba* and *atp6v1B2* [ZFIN]^15^, have also been reported in teleost fish^16^. It has been postulated that they are, respectively, orthologs of the tetrapods *atp6v1B1* and *atp6v1B2*^17^. So far, no mutations have been described for these subunits in teleosts.

In this study we describe the identification, cloning and analysis of the medaka (*Oryzias latipes*) mutant *tintachina* (*tch*), which carries a loss-of-function mutation in *atp6v1Ba/vatB1*. The mutation causes pigmentation defects and brain degeneration and involves the missense modification (G75R) of a glycine residue, conserved across eukaryotes, to arginine. An equivalent homozygous mutation in *atp6v1B1* (G78R) has been reported in a family affected with dRTA^18^. This serendipitous coincidence allows comparison of the physiological consequences of an equivalent loss of function in genes that share a common ancestor. Here we analyze the phylogenetic relationship between the different vertebrate B subunits and offer a hypothesis on their evolutionary history and their divergent functional adaptations.

## Results

### The *tintachina* mutation disrupts *atp6v1Ba/vatB1*

**Figure 1:**
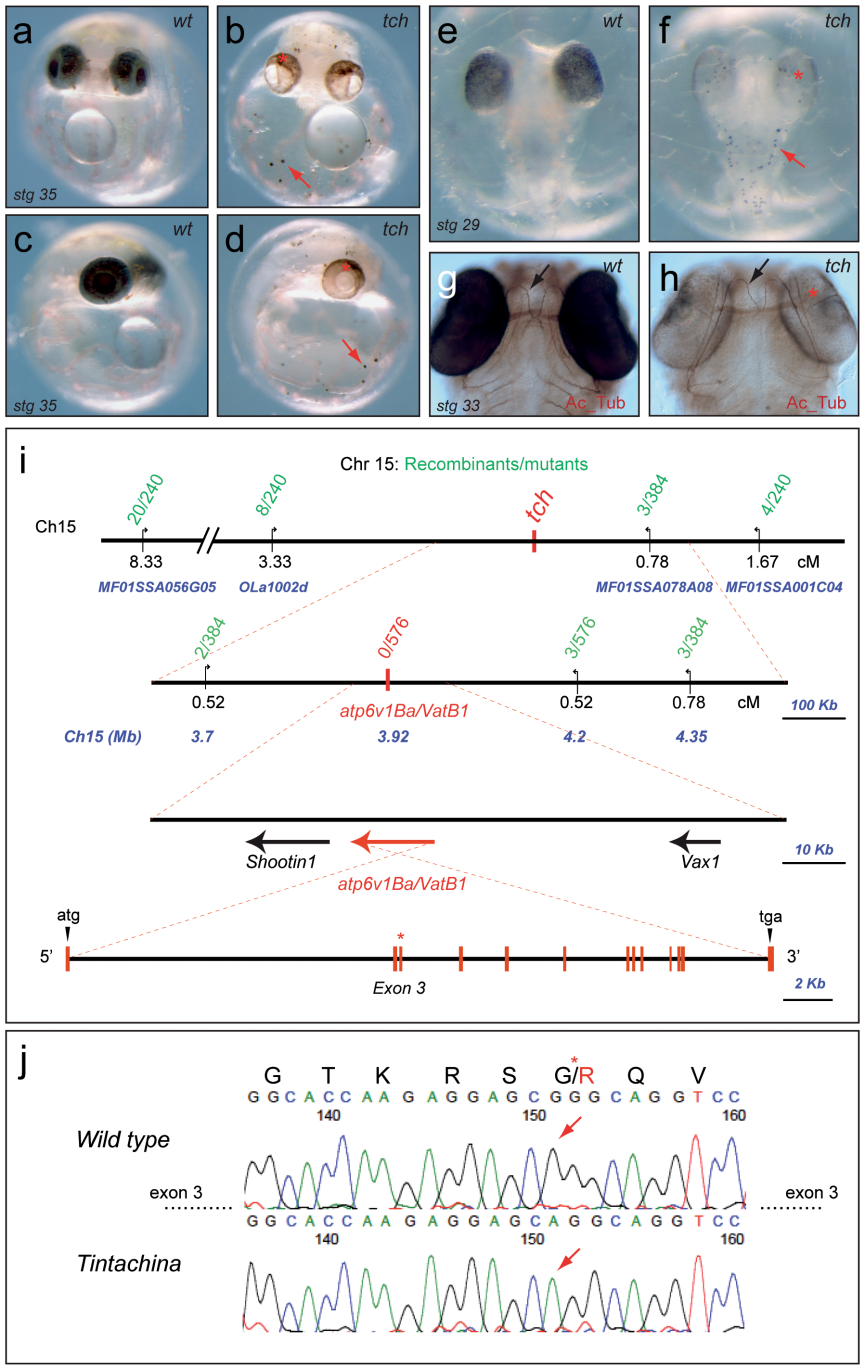
*Tch* phenotype and positional cloning. (a–d) Comparison of wt (a,c) and *tch* (b,d) medaka embryos at stage 35 shows reduced pigmentation in the mutants. (e–f) Mutant phenotype becomes first apparent at stage 29. Red arrows and asterisks indicate hypopigmented eyes and characteristic dotted melanocytes respectively. (g–h) Acetylated tubulin staining of wt (g) and *tch* (h) stage 33 embryos reveals normal axon scaffold organization (arrows). (i) Genetic and physical map of the medaka *tch* locus on chromosome 15. Recombinants are indicated in green above the chromosome line. Genetic (cM) and physical (Kb) distances are indicated below. Transcription units are depicted as arrows. Chromosomal markers used for fine mapping are indicated in blue. The genomic structure of *atp6v1Ba* is depicted: exons are represented as red bars. The mutated exon 3 is indicated with an asterisk. (j) The sequencing trace data from wild-type and *tch* mutants cDNA and their predicted translation is depicted. Sequencing reveals a G to A point mutation (arrows).

In an ENU (*N*-ethyl-*N*-nitrosourea) mutagenesis screen for mutations affecting retina development in medaka^19^ we identified the mutation *tintachina* (*tch*), which displayed reduced pigmentation of the eye and punctate body pigmentation (Fig. 1a–d). The mutation was named *tintachina* (‘chinese ink' in Spanish) after the characteristic melanocyte pattern and has been transmitted through more than 12 generations without noticeable phenotypic changes. It is a lethal recessive mutation that shows full penetrance and minimal phenotypic variability. The mutant phenotype first becomes apparent as pigmentation emerges between stages 28–29 by reduced pigmentation of the eyes (Figure 1e, f). At early organogenesis, no morphogenetic defects are observed in *tch* embryos, which show normal organization of body plan and axon scaffolds, as assessed by anti-acetylated tubulin labeling (Figure 1g, h). At later stages, mutant embryos suffer progressive tissue degeneration, particularly in the CNS, and finally die between stages 37 to 39, shortly before hatching.

We mapped the *tch* locus to chromosome 15 by bulk segregation analysis^20^. Further mapping reduced the region of interest to an interval of 700 kbp, as defined by two flanking restriction length polymorphisms (RFLPs), which contained a few candidate genes including *atp6v1Ba/vatB1* (Figure 1i). The characteristic *tch* phenotype: hypopigmentation of the eyes, punctate melanocytes, and progressive brain degeneration, has been described in a number of zebrafish mutants affecting different subunits of the vacuolar proton pump including *atp6v0d1*, *atp6v0c*, *atp6v1H*, *atp6v1F* and *atp6v1E1*^21^,^22^,^23^. Therefore, we decided to investigate whether the *tch* mutation was associated with *atp6v1Ba/vatB1*. A RFLP analysis of the *atp6v1Ba/vatB1* locus showed no recombinant chromosomes (0/576) in the mutant embryos (Figure 1i), thus suggesting that it was the mutated gene. To confirm this, the entire coding region of *atp6v1Ba/vatB1* was amplified by PCR from cDNA and sequenced in several independent wild type and *tch* embryos. A missense mutation altering glycine to arginine at position 75 (G75R) was consistently identified in mutant embryos (Figure 1j). This missense mutation was further confirmed by sequencing the genomic region encompassing exon 3 in wild type and *tch* embryos. The G75R point mutation (-RS-**G/R-**QVLE-) lies within a highly conserved domain (Supplementary Figure S1) in a glycine residue preserved in all metazoans and even in other eukaryotes, such as the yeast *Saccharomyces*. A L81P point mutation (-RSGQV-**L/P**-E-) in the same conserved domain of *atp6v1B1* has been identified as causative for dRTA in humans^7^. Moreover, an equivalent homozygous mutation (G78R: -RS-**G/R-**QVLE-) caused by the same nucleotide substitution (g/a) has been reported in a Turkish family affected with dRTA^18^.

### Lysosomal function and neuronal survival are compromised in *tch*

**Figure 2:**
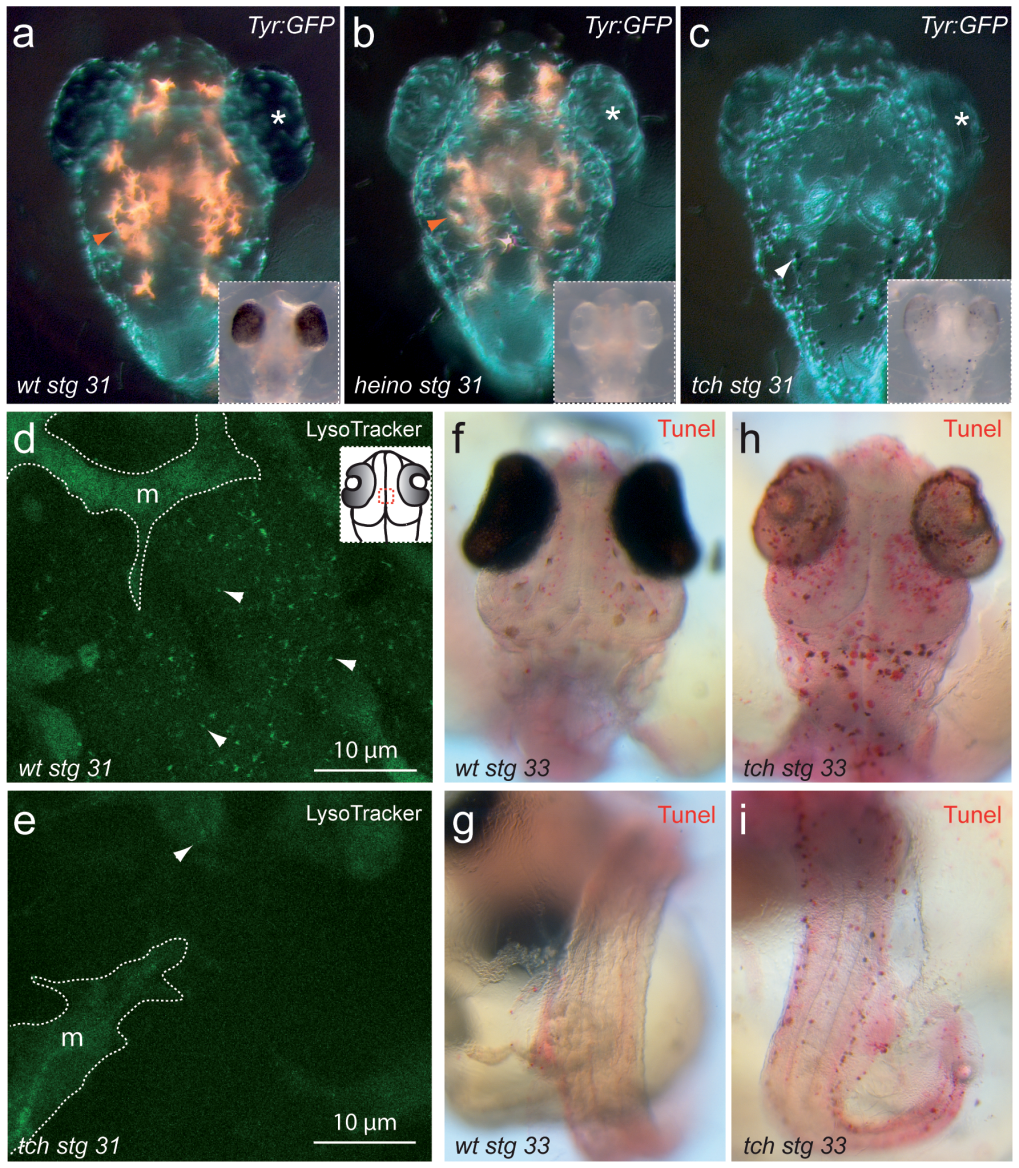
Lysosomal function and neuronal survival are compromised in *tch*. (a–c) Melanophores, as revealed by the transgenic line *Tyr::GFP* (green), and auto-fluorescent xantophores (orange) are shown in wild type (a), *heino* (b) and *tch* (c) embryos at stage 31. Eye pigmentation defects (asterisk) are shown in fluorescent and transmitted light images (insets). White arrow in “c” points to dotted melanocytes in tch. (d–e) LysoTracker Green *in vivo* labelling reveals acidic organelles (arrows) at the dorsal diencephalon (see inset in d) in wild type (d) and *tch* (e) stage 31 embryos. *Tyr::GFP* positive melanophores (m) are indicated with dotted lines as an internal reference. (f–i) Tunel staining shows apoptotic cells in wt (f,g) and *tch* (h,i) medaka embryos at stage 33 in the anterior brain (f,h) dorsal neural tube and fin folds (g,i).

Oculocutaneous albinism is a common trait in congenital disorders affecting different aspects of pigment cell biology. Anomalies range from melanophore specification/migration defects to abnormal melanosome maturation and melanin synthesis. To analyze at which level *tch* embryos are affected, we examined the mutants in the background of the transgenic line *Tyr::GFP*, which labels differentiated melanophores, and to a lesser extent xanthophores^24^. As a parallel control we crossed the viable albino mutation *heino*^25^, which also affects melanin synthesis, into the same *Tyr::GFP* line. A normal distribution and number of GFP-positive cells were observed in *tch* and *heino* (Figure 2a–c), thus indicating that melanophore migration and differentiation is unaffected in both mutants. This observation is in line with previous reports showing that V-ATPase function is required for both melanosome maturation and melanin synthesis^22^. In addition, the orange auto-fluorescent sepiapterins, synthesized in xantophores in acidic organelles homologous to the melanosomes^24^, appear to be completely absent in *tch* (Figure 2c). This suggests a more general requirement for *atp6v1Ba/vatB1* in the acidification and biogenesis of lysosomal-related acidic organelles. To further investigate this, we labeled live embryos with LysoTracker Green, a probe that selectively accumulates in acidic intracellular compartments. After a 10 min pulse, LysoTracker accumulation was imaged in the dorsal diencephalon of stage 31 *tch* embryos and wild type siblings. Whereas the probe was significantly incorporated into acidic compartments in wild type cells, a very limited accumulation was observed in *tch* (Figure 2d, e), thus indicating that lysosomal acidification is compromised in the mutants.

Previous reports have shown that lysosomal dysfunction mediated by V-ATPase inhibition induces apoptosis in mammalian cells^26^,^27^. Conversely, the overexpression of atp6v1B2 in HEK cells increased their resistance to apoptosis^28^. To further understand the progressive degeneration observed in *tch* embryos, we investigated by TUNEL assay whether this was due to an enhanced apoptotic rate. Whole mount TUNEL staining in stage 33 embryos revealed increased apoptosis in *tch*. In the mutants, apoptotic cells were particularly detected in the retina, diencephalon and mesencephalon (Figure 2 f–h) and to a lesser extent in the dorsal neural tube, ventral mesoderm and fin folds (Figure 2g–i). Therefore, we confirmed a role for *atp6v1Ba/vatB1* as a regulator of lysosomal function and neuronal survival.

### The evolution of V-ATPase B subunits within the vertebrate group

**Figure 3:**
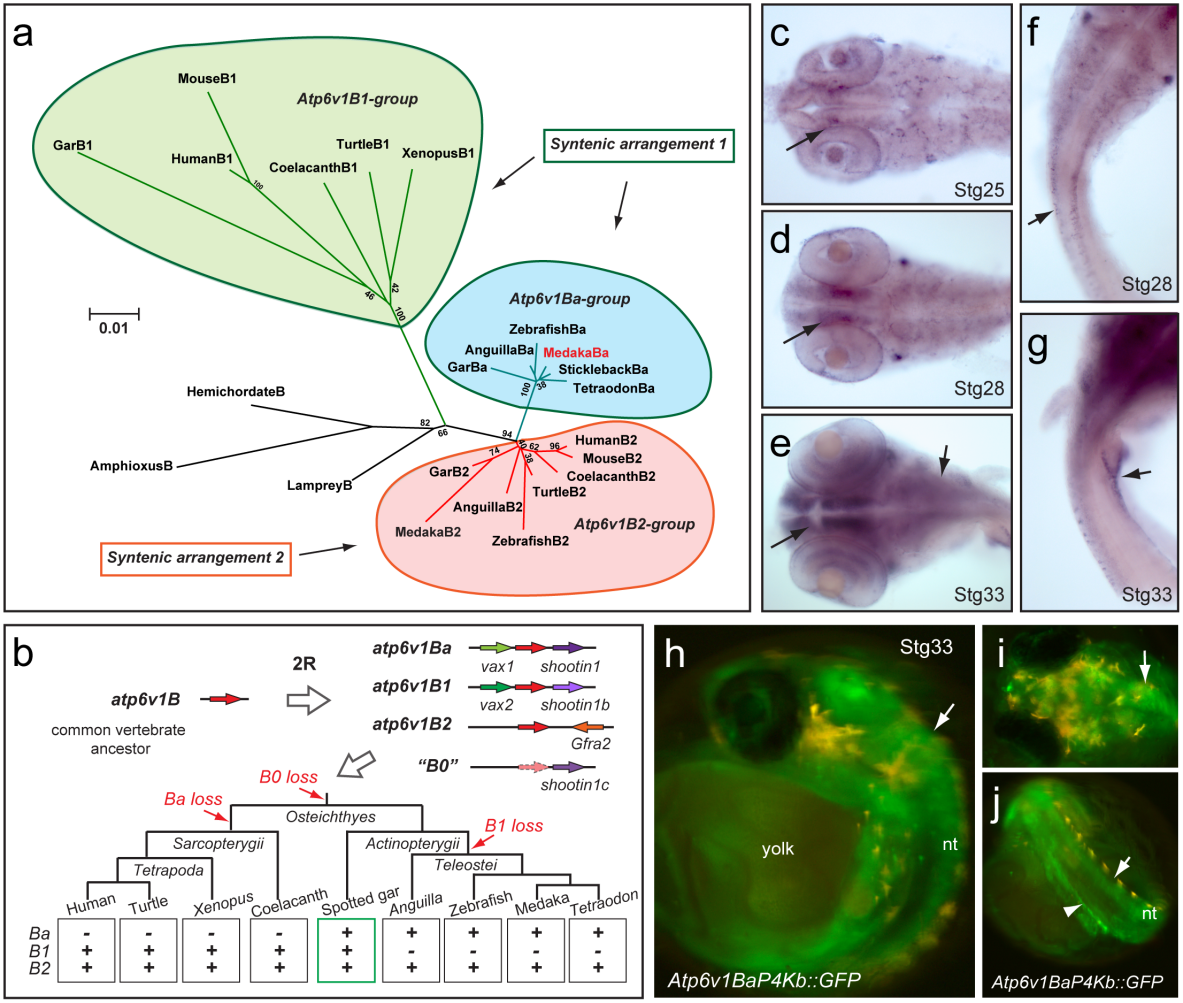
The evolution of V-ATPase B subunits within the vertebrate group. (a) Phylogenetic analyses defined three different B subunits groups (here color-coded). The syntenic arrangement of the different genes is indicated. See also Supplementary Figure S1 and Table 1. (b) Schematic representation of the evolutionary history of atp6v1B genes in vertebrates. Whole genome duplications and *atp6v1B* gene loss events at the different lineages under the most parsimonious scenario are depicted. See also Supplementary Figures S3 and S4. (c–g) *Atp6v1Ba* expression in medaka embryos revealed by whole-mount ISH at stages 25 (c), 28 (d, f) and 33 (e, g). Arrows point to specific expression in the diencephalon (c–e), dorsal neural tube (f) and pronephros (g). (h–j) *Atp6V1BaP4Kb::GFP* expression in stage 33 embryos. Arrows point to specific expression in the nervous system (h, i) and the pronephros (j).

It has been postulated that *atp6v1Ba/vatB1* is the true teleost ortholog of the mammalian gene *atp6v1B1*^17^. To confirm this point and to further investigate the evolutionary history of the *atp6v1B* family, we identified and analyzed *atp6v1B* genes in vertebrate and invertebrate genomes. Multiple sequence alignments revealed that vertebrate B subunits are highly conserved, showing an average % of aminoacid identity of 86.9±0.4 (n = 20; mean±SEM) when compared to *Drosophila* atp6V1B (Supplementary Figure S1). We then generated a protein family tree using a maximum likelihood approach (Figure 3a). In contrast to what would be expected for orthologous genes, teleost atp6v1Ba/vatB1 and mammalian atp6v1B1 proteins did not cluster together in the tree. In fact, phylogenetic analyses defined three different B subunit monophyletic groups: an atp6v1B2 group that includes B2 subunits from all studied jawed vertebrate lineages (average % of identity with *Drosophila* 87.8±0.7); an atp6v1Ba group that includes teleost proteins (average % of identity with *Drosophila* 87.1±0.1), and finally a somewhat more divergent atp6v1B1 group comprising of tetrapod and coelacanth proteins (average % of identity with *Drosophila* 85.1±0.5).

Teleost genomes share an extra third round of whole genome duplication (or 3R) that occurred at the root of the lineage^29^ and may obscure orthology assignment when genes in tetrapods and teleosts are compared. To investigate this possibility, we studied the genome of the spotted gar (*Lepisosteous oculatus*), a basal ray-finned fish that diverged from teleosts before the occurrence of the 3R event^30^,^31^. Importantly, we found gar representatives for each of the three paralogs: *Ba*, *B1* and *B2* (Figure 3a). This suggests that *Ba* genes are not divergent duplicated teleost orthologs of any of the tetrapod genes, but ancient jawed vertebrate paralogs that emerged after the first two rounds of genome duplication (2R) that happened during the evolution of vertebrates^32^,^33^. Subsequently, *B1* and *Ba* would have been selectively lost in all the studied species of the teleost and sarcopterigian (tetrapods and coelacanth) lineages respectively (Figure 3b). In fact, divergent *Ba* pseudogenized remnants are still detectable in sarcopterigian species such as coleacanths, painted turtles and humans (Supplementary Figure S2). Therefore, our phylogenetic analyses indicate that the medaka atp6v1Ba/vatB1 protein belongs to the atp6v1Ba clade (Figure 3a). Consistently, in situ hybridization analysis of *atp6v1Ba* expression in medaka embryos (Figure 3c–g) showed a very similar pattern to that reported for the zebrafish *atp6v1Ba* ortholog in the database ZFIN^15^. In early organogenesis stages, *atp6v1Ba* could be detected at low levels in most medaka tissues (Figure 3c). As development proceeds, *atp6v1Ba* transcripts accumulation was progressively detected in the central nervous system; particularly in the diencephalon, retina and dorsal neural tube (Figure 3c–f). An additional expression domain was also detected in the anterior pronephros at stage 33 (Figure 3g). *Atp6v1Ba* complex expression is in contrasts with the more restricted *atp6v1B1* expression in mammals. In fact, it rather resembles *atp6v1B2* expression, which was initially described as an isoform ubiquitously expressed at low levels in a range of human tissues, but at significantly higher levels in brain and kidney^12^.

### Syntenic analysis of the *atp6v1B* gene family

To complement our phylogenetic studies we examined synteny conservation between vertebrate genomes around the *atp6v1B* paralogous loci and between the equivalent regions in two non-vertebrate deuterostomes: the basal chordate amphioxus (*Branchiostoma floridae*) and the hemichordate *Saccoglossus kowalevskii* (Supplementary Figure S3). Extensive conservation across species in this genomic neighborhood further demonstrated the orthology relationships previously uncovered by the phylogenetic analysis, fully supporting the existence of three duplicated *atp6v1B* genes in the last common ancestor of bony vertebrates. As in the case of the B subunits, many of the genes located in their vicinity (e. g. *Shootin*, *Slc18a* and *Gfra*) were also present in several copies in most of the studied genomes. Consistent with their origin at the vertebrate whole genome duplication events, the location of most of these duplicates is confined to the same set of paralogous chromosomal segments where the *atp6v1B* genes are located: 10q26, 2p13 and 8p22 in the human genome (Supplementary Figure S3). Furthermore, some of the copies were located in an additional chromosomal segment, 5q31-33 in humans, raising to four the number of paralogous chromosomes derived from the same ancestral linkage group, in full agreement with the general pattern of quadruple conserved macrosynteny typical of vertebrate genomes^33^. This implies that a fourth *atp6v1B* gene was present in the ancestral jawed vertebrate after the 2R events and was subsequently lost before bony vertebrate radiation.

We then compared the syntenic arrangement of the three paralogous regions (Ba, B1 and B2) containing *atp6v1B* genes plus the fourth region, currently devoid of an *atp6v1B* copy (hereafter B0), to infer their evolutionary relationships. Three different topologies are possible: [Ba-B1, B2-B0], [Ba-B2, B1-B0] and [Ba-B0, B1-B2]. Among these, the [Ba-B0, B1-B2] grouping was not consistent with any differentially shared syntenic arrangement, as it would imply more gene losses than the other two scenarios. The other two topologies, [Ba-B1, B2-B0] and [Ba-B2, B1-B0], were both supported by the presence of differentially shared duplicated genes (Supplementary Figure S4). Although the arrangement [Ba-B1, B2-B0] may seem somewhat more likely, any hypothesis should be interpreted with caution as gene loss is a pervasive phenomenon in evolution, especially in the context of the genetic redundancy created by whole genome duplications^34^. In either case, both topologies indicate that *atp6v1Ba* and *atp6v1B1* are paralogous genes radiating from a common ancestor that lay in close proximity to a *vax* gene (Supplementary Figure S4).

### Cis-regulatory sub-functionalization within the *atp6v1B* gene family

In vertebrates, sub-functionalization of paralogs after gene duplication has frequently occurred by loss or modification of *cis*-regulatory modules^35^. The differential expression pattern observed between *atp6v1Ba* and *atp6v1B1* indicates that such a process may have taken place, resulting in divergent, tissue specific, functions. Through transgenesis in mice, it has been shown that a 6.5 kb fragment of the *atp6v1B1* promoter (comprising the entire intergenic region between *atp6v1B1* and *vax2*) contains enough regulatory information to recapitulate the specific expression of the gene in the kidney, lung and epididymis^14^. Interestingly, this intergenic region between *atp6v1B1* and *vax2* comprises of only a few kb in tetrapods, while the equivalent region between *atp6v1Ba* and *vax1* expands over 10–20 kb in more compact fish genomes (Table 1). This suggests that 5′ *cis*-regulatory elements, responsible for the widespread expression of the gene, may have been lost at the *atp6v1B1* locus after genome duplication. According to this hypothesis, such elements would have been retained in the intergenic region between *atp6v1Ba* and *vax1*. To test this possibility we analyzed the regulatory information contained in the 5′ region of the medaka *atp6V1Ba*. A 4 kb fragment upstream of the gene was fused to GFP and injected into medaka embryos to generate stable reporter lines (*Atp6V1BaP4Kb::GFP*). The expression pattern driven by this element was already visible in F0 embryos and was further consistently confirmed in F1 for three independent insertions. In agreement with *atp6V1Ba* expression, GFP signal was weakly detected at early stages in medaka embryos (not shown) and increased later in development, accumulating mainly in the central nervous system and pronephros at stage 33 (Figure 3h–j). This finding indicates that the intergenic region between *atp6v1Ba* and *vax1* contains sufficient *cis*-regulatory information to recapitulate the widespread, and likely ancestral, expression of the gene.

## Discussion

Here we report the identification and analysis of the mutation *tch*, a missense mutation (*G75R*) of the proton pump subunit *atp6v1Ba* that causes oculocutaneous albinism, progressive brain degeneration and embryonic lethality. Our analyses indicate that *atp6v1Ba* plays a role in the acidification of intracellular compartments and hence its lack of function induces apoptosis in those tissues in which it is normally expressed. Consistent with the essential role of V-ATPase complexes in organelle acidification, mutations in almost any of its subunits cause embryonic lethality^11^,^22^,^23^. Exceptions to this are those subunits involved in intracellular targeting, such as the mammalian *atp6v1B1*, which shows a tissue-restricted distribution and does not play an essential role during embryogenesis^9^. In this study we have presented evidence showing that the teleost gene *atp6v1Ba/vatB1* is not the real ortholog of the mammalian gene *atp6v1B1*, as previously postulated^17^; on the contrary, we show that *atp6v1Ba* and *atp6v1B1* are paralogous genes which emerged after whole genome duplication events. Despite the fact that both proteins share high sequence similarity (>80% of identity) and play a conserved molecular function, their mutations result in fundamentally different phenotypes. Although subtle changes in protein sequence could partially explain this discrepancy, it is likely that *atp6v1Ba* and *atp6v1B1* divergent expression patterns have a major impact in their physiological role, thus accounting for a large part of the phenotypic variation observed when mutated. In line with this, our data suggest that gene duplication within the vertebrate *atp6v1B* family has allowed the divergence of their *cis*-regulatory modules. This process may have relieved evolutionary constraints, allowing *atp6v1B1* to acquire tissue-restricted expression. In turn, specific expression would facilitate the adaptation of the protein from a lysosomal/endosomal function to a role at the plasma membrane. At the physiological level, the membrane targeting of the B1 subunit represented an important adaptation in the context of the functional requirements of the mammalian kidney^4^ or the amphibian larval skin^36^.

## Methods

### Fish stocks and transgenic lines

Medaka (*Oryzias latipes*) strains Cab and Kaga were kept as described previously^19^. The Kaga strain was used for chromosomal assignation and positional cloning. The stable *Tyr::eGFP* line has been previously described^37^. To generate the stable *atp6V1Baprom4Kb::GFP* line using I-SceI mediated transgenesis, a fragment of 4 Kb upstream of the atg of the medaka *atp6V1ba* gene was amplified and fused to eGFP in a meganuclease compatible vector^38^. The coordinates of this fragment in the medaka genome are the following: Ch15: 3952227-3956219.

### Genetic mapping and positional cloning

*tch* was assigned to chromosome 15 by bulk segregation analysis using the Kaga strain as a reference^20^. The genetic distance to the locus was narrowed by available genetic markers^39^ on 576 mutant chromosomes. We used the markers Ola1002d (8/240; 3.3 cM) and MF01SSA078A08 (3/384; 0.78 cM) as initial references flanking the mutation. From these anchoring positions, new restriction length polymorphisms were designed and examined as nested reference points through the chromosome. The amplified medaka *atp6v1Ba* full-length sequence and the 3′ terminal fragment (377 bp) of the medaka *atp6v1B2* have been deposited in GeneBank (JX416286 and JX416287 respectively).

### LysoTracker staining

Embryos were dechorionated with Proteinase K (10 mg/ml in H_2_O) and hatching enzyme as described^37^. Stage 31 dechorionated embryos were incubated for a 10 min pulse in 1 μM LysoTracker Green (DND26; Invitrogene) at 25°C and then imaged by confocal microscopy using a Leica SP5 system.

### Whole-mount acetylated tubulin staining

were performed as follows: embryos were fixed with 20% DMSO/80% MeOH at room temperature for 2 hours, treated with 10% H_2_O_2_/10% DMSO/80% MeOH overnight at 4°C, and incubated with anti-acetylated tubulin (Sigma T6793) monoclonal antibodies at 1:1000 dilution. After incubation with peroxidase-coupled secondary anti-mouse antibodies (1:2000 Sigma) samples were stained using a NovaRed substrate kit (Vector Laboratories).

### Whole-mount in situ hybridization

Whole-mount in situ was performed using digoxigenin labeled riboprobes as described^37^.

### Whole mount tunel staining

Embryos were with fixed with 4% p-formaldehyde in PBS overnight at 4°C, stored in 100% methanol, and then rehydrated in PBS-tween 0.1% (PBT) before treatment with proteinase k (0.2 mg/ml in PBT) for one hour. After post-fixation with 4% p-formaldehyde apoptotic cells were identified using the in situ cell death detection kit AP (Roche). Alkaline phosphatase associated signal was visualized with FastRed (Roche).

### Search for *atp6v1B* genes, phylogenetic analysis and genome browsing

In species where *atp6v1B* genes were not previously identified or available, or where gene predictions were fragmentary or poorly annotated, we built new manually curated predictions as described previously^40^. In the case of medaka and *Tetraodon* *atp6v1B2* genes some gaps/Ns (indicated by “X” in Supplementary Figure 1) have to be included in order to maintain an ancestral exon/intron boundary and the reading frame near the translation start codon, respectively. This suggests that these genes could be subjected to an ongoing pseudogenization process. We aligned atp6v1B proteins using MAFFT^41^ as implemented in Jalview 2.8^42^, and discarded poor quality regions (the N- and C- terminal portions, the first 29 and the last 23 positions in the alignment of Supplementary Figure 1) from the alignment for subsequent phylogenetic analysis. We built maximum likelihood trees with MEGA 5^43^, under the most complex available model (WAG + I + Γ) and 100 bootstrap replicates. Very divergent sequences, such as those of fly, sea squirt, and frog atp6v1B2 were excluded from phylogenetic analysis.

Genomes of the studied species were browsed through the JGI (http://genome.jgi-psf.org/euk_home.html), NCBI (http://www.ncbi.nlm.nih.gov/blast/Blast.cgi), UCSC (http://genome.ucsc.edu/) and Ensembl (http://www.ensembl.org/info/about/species.html) webpages, using the following genome versions: *Branchiostoma floridae* v1.0, *Chrysemys picta* v3.0.1, *Ciona intestinalis* v2.0, *Danio rerio* Zv9, *Drosophila melanogaster* R5.48, *Gasterosteus aculeatus* 1.0, *Homo sapiens* Build 37, *Latimeria chalumnae* v1, *Lepisosteus oculatus* v1, *Mus musculus* Build 38, *Oryzias latipes* 2.0, *Petromyzon marinus* v7.0, *Saccoglossus kowalevskii* Build1.1, *Tetraodon nigroviridis* v8 and *Xenopus* *tropicalis* v4.1. Eel atp6v1B gene complement was inferred from the Japanese eel (*Anguilla japonica*) genome downloaded from the eel genome website (www.eelgenome.com/). For the alignment and phylogenetic analyses, full length protein sequences of the European eel (*A. anguilla*) *atp6v1Ba* and *atp6v1B2* orthologs (previously published under accession numbers AAD55091 and AAC78641, respectively) were used.

## Author Contributions

Most of the experimental work was done by C.M. and J.R.M.-M. in J.W.'s and J.R.M.-M.'s laboratories. I.M. contributed with key phylogenetic analyses. The manuscript was written by J.R.M.-M.

## Supplementary Material

Supplementary Information Supplementary information

## Figures and Tables

**Table 1:** Size (in kb) of the *atp6v1B-vax* intergenic region in different vertebrate species

Species	Gene name	Ensembl ID	Chromosome/scaffold	Distance to *Vax* (Kb)
Human	*atp6v1B1*	ENSG00000116039	Ch2	2.5
Mouse	*atp6v1B1*	ENSMUSG00000006269	Ch6	4.6
Xenopus (tropicalis)	*atp6v1B1*	ENSXETG00000021532	Scaffold_GL173004.1	5.5
Anole Lizard	*atp6v1B1*	ENSACAG00000007860	Scaffold_GL344660.1	nd
Coelacanth	*atp6v1B1*	ENSLACG00000003890	Scaffold JH128355.1	nd
Spotted Gar	*atp6v1B1*	PreEnsmbl(NA)	Chr4:2227818-2264652	4.9
Spotted Gar	*atp6v1Ba*	PreEnsmbl(NA)	Ch5:19273911-19315700	9.9
Zebrafish	*atp6v1Ba*	ENSDARG00000013443	Ch17	14.4
Medaka	*atp6v1Ba*	ENSORLG00000001220	Ch15	16.5
Stickleback	*atp6v1Ba*	ENSGACG00000014640	Scaffold_76	22.9
Tetraodon	*atp6v1Ba*	ENSTNIG00000005817	Un_random:	10.6
