# Supplementary material for: The medaka mutation tintachina sheds light on the evolution of V-ATPase B subunits in vertebrates
Source: Sci Rep. 2013 Nov 14;3:3217. doi: 10.1038/srep03217 (PMC3827601; doi:10.1038/srep03217)
Supplement: Supplementary Information [file srep03217-s1.pdf]

Supplemental Online Material

The medaka mutation *tintachina* sheds light on the evolution of V-ATPase B subunits in vertebrates.

Claudia Müller [2*], Ignacio Maeso[3*], Joachim Wittbrodt [2#], Juan R. Martínez-Morales[1#*]

[#]Correspondence: jochen.wittbrodt@cos.uni-heidelberg.de; jrmarmor@upo.es

Supplemental Data includes: 4 Figures:

Figure S1. **MAFFT multiple alignment of vertebrate and invertebrate *atp6v1B* subunits**
Key amino acids mutated in human patients (L81P and G78R in *atp6v1B1)* and in medaka *tch* embryos (G75R in *atp6v1Ba*) are indicated in red. Flanking amino acids are shaded in a grey box. Manually curated amino acids are indicated in blue. See also Figure 3.

Figure S2. Atp6v1Ba **pseudogenic remnants in sarcopterygians**
Nucleotide (upper rows) and aminoacid (lower rows) alignments of the pseudogenic exons 7 (A) and 12 (B) from coelacanth, human and painted turtle, and the corresponding orthologous intact exons from gar. Splice donors (gt) and acceptors (ag) are represented in lower case. Frameshift, stop codon or splice site mutations are depicted in red and indicated with “X” (frameshifts) or asterisks (stop codons).

Figure S3. **Conserved synteny around *atp6v1B* loci**
Schematic view of the genes (arrows) surrounding *atp6v1B* loci (red arrows) in two invertebrate deuterostomes (A) and their correspondent vertebrate co-orthologues *Ba* (B), *B1* (C), *B2* (D) and “*B0*” (E). The names of *atp6v1B* neighbouring genes conserved between vertebrates and invertebrates are labelled in red. Genes conserved between at least two paralogous vertebrate chromosomes are coloured. Dotted lines in the arrows corresponding to human and coelacanth *atp6v1Ba* genes indicate their pseudogenic nature. Slashes represent genomic intervals where the gene content has not been detailed in the figure. Question marks correspond to genes with no clear orthology relationships and solid circles indicate the end of the corresponding chromosome/scaffold. Note that in the case of human *B1* genomic region, the ancestral syntenic segment has been secondarily separated into two different chromosomes, 2 and 20. Intergenic distances are not drawn to scale.

Figure S4**. Proposed evolutionary history of *atp6v1B* gene family**
The syntenic arrangement surrounding *atp6v1B* genes in modern vertebrate groups is outlined in panel A. Two different hypotheses explaining the paralogy relationships between modern *atp6v1B* genes are depicted. Same colour code was used to represent conserved genes. The topologies [Ba-B1, B2-B0] and [Ba-B2, B1-B0], both supported by the presence of differentially shared duplicated genes (see also Fig S3), are schematized in panels B and C respectively.

MAFFT multiple alignment

```
DrosophilaB        ----------------------------MNAQQAQREHVLAVSRDFISQPRLTYKTVSGV
HemichordateB      -----------------MNGDLMDLHSSKNNLQASREHALAITRNYISQPRLTYKTVSGV
AmphioxusBa        -------------MSAMGDLMDLGSTPPTNDIRMAREHTLAITRNYHSQPRLTYKTVTGV
CionaB             ---------------MNGDLMQMTDALSNATLAGAREHTLAVTRDYISQPRLTYQTVSGV
LampreyB           ------------------------------------------------------------
AnguillaB1(Ba)     ----------------MATLVENRNVELNGPEAAARQHAQAVSRNYISQPRLTYSTVSGV
ZebrafishBa        ----------------MSTLVANRPVDLNGPEAAARQHAQAVVRNYISQPRLTYTTVSGV
GarBa              ---------------MATMVGSRPIDTLNGPEAAARQHAQSVTRNYISQPRLTYSTVSGV
MedakaBa           ----------------MATLVANRGMDVNGLAGAARTHTQAVTRNYISQPRLTYSTVSGV
SticklebackBa      ----------------MATLVANRAMDINGLAGAARTHTQAVTRNYISQPRLTYSTVSGV
TetraodonBa        ----------------MATLVANRPMEVNSLAAGARTHTQAVTRNYISQPRLTYSTVSGV
MouseB2            -------MALRAMRGIVNGAAPELPVPTGGPMAGAREQALAVSRNYLSQPRLTYKTVSGV
HumanB2            -------MALRAMRGIVNGAAPELPVPTGGPAVGAREQALAVSRNYLSQPRLTYKTVSGV
CoelacanthB2       -------MALKAIRGMVNGAMSEITSTVS-SAAGAREHVQAVSRDYISQPRLTYKTVSGV
XenopusB2          -------MAVRVMRGGEGGPG-------NPIGAAAREHVQAVSRDYLSQPRLTYRTVSGV
GarB2              MALKAIRGMVNGAMNELSSTVSG------TKAAGAREHVQAVTRDYISQPRLTYKTVSGV
AnguillaB2         MAMKAIRGIVDGAMTELSSAVSG------TKAAMTREHVQAVSRDYISQPRLTYKTVSGV
ZebrafishB2        --MKALRGMVSGAVSEISSSLTG------TKQVAVRENVLAVTRDYISQPRLTYKTVAGV
TetraodonB2        MAMKALRGMVDGAMSELSSAVGGAPPPAAAAAAAAREQVLAXXEDHRSQPRLTYTTVSGV
MedakaB2           -----------------------------------------------------YKTVSGV
MouseB1            -------------MATTVDSRSSGFTGNSCDPGTAQEHVQAVTRNYITHPRVTYRTVCSV
HumanB1            -------------MAMEIDSRPGGLPGSSCNLGAAREHMQAVTRNYITHPRVTYRTVCSV
CoelacanthB1       -----------------------------------------------------YRTVCGV
XenopusB1          ----------------------MRVEGHQANMAAAREHMMAVTRNYISQPRLSYRTVCGV
GarB1              ---------------------MSRQAETRTRVAAAREHALAVSRDYISQPRTTYRTVCGV


DrosophilaB        NGPLVILDEVKFPKFAEIVQLRLADGTVRSGQVLEVSGSKAVVQVFEGTSGIDAKNTLCE
HemichordateB      NGPLVILDDVKFPKYAEIVTLTLADGTQRSGQVLEVSGSKAVVQVFEGTSGIDAKHTTVE
AmphioxusBa        NGPLVILDDVKFPKYAEIVHLTLNDGSIRSGQVLEVSGSKAVVQVFEGTSGIDAKHTTCE
CionaB             NGPLVILDKVKFAKYAEIVTLNLADGTQRSGQVLEVSGEKAIVQVFEGTSGIDAKYTTCE
LampreyB           -------------------------------------------QVFEGTSGIDARKTTCE
Anguilla(Ba)       NGPLVILDNVKFPRYAEIVHLTLPDGTKRSGQVLEVIGSKAVVQVFEGTSGIDAKKTACE
ZebrafishBa        NGPLVILDNVKFPRYAEIVHLTLPDGTKRSGQVLEVIGTKAVVQVFEGTSGIDAKKTACE
GarBa              NGPLVILDNVKFPRFAEIVHLTLPDGTKRSGQVLEVMGSKAVVQVFEGTSGIDAKKTACE
MedakaBa           NGPLVILDNVKFPRYAEIVHLTLPDGTKRSGQVLEVIGTKAVVQVFEGTSGIDAKKTACE
SticklebackBa      NGPLVILDNVKFPRYAEIVHLTLPDGTKRSGQVLEVIGSKAVVQVFEGTSGIDAKKTACE
TetraodonBa        NGPLVILDNVKFPRYAEIVHLTLPDGTRRSGQVLEVIGSKAVVQVFEGTSGIDAKKTACE
MouseB2            NGPLVILDHVKFPRYAEIVHLTLPDGTKRSGQVLEVSGSKAVVQVFEGTSGIDAKKTSCE
HumanB2            NGPLVILDHVKFPRYAEIVHLTLPDGTKRSGQVLEVSGSKAVVQVFEGTSGIDAKKTSCE
CoelacanthB2       NGPLVILDQVKG------------------------------------------------
XenopusB2          NGPLVILDNVKFPRYSEIVHLTLPDGTKRSGQVLEVSGSKAVVQVFEGTSGIDAKKTSCE
GarB2              NGPLVILDQVKFPRYAEIVHLTLPDGTRRSGQVLEVSGSKAVVQVFEGTSGIDAKKTSCE
AnguillaB2         NGPLVILDHVKFPRYAEIVHLTLPDGTKRSGQVLEVSGSKAVVQVFEGTSGIDAQKTSCE
ZebrafishB2        NGPLVILDQVKFPRYAEIVHLTLPDGTKRSGQVLEVTGSKAVVQVFEGTSGIDAKKTTCE
TetraodonB2        NGPLVILDQVKFPRYAEIVHLTLPDGTRRSGQVLEVTGSKAVVQVFEGTSGIDAKKTSCE
MedakaB2           NGPLVILDQVKFPRYAEIVHLTLPDGTSRSGQVLDVNGSKAVVQVFEGTAGIDAKKTSCE
MouseB1            NGPLVVLDQVKFAQYAEIVNFTLPDGTQRSGQVLEVAGTKAIVQVFEGTSGIDSQKTTCE
HumanB1            NGPLVVLDRVKFAQYAEIVHFTLPDGTQRSGQVLEVAGTKAIVQVFEGTSGIDARKTTCE
CoelacanthB1       NGPLVVLDNVKFAKYAEIVHFTLPDGIMRSGQVLEVVGSKAIVQVFEGTSGIDAKKTTCE
XenopusB1          NGPLVVLDNVKFAQYAEIVNFTLPDGTSRSGQILEVSGSKAIVQVFEGTSGIDAKKTTCE
GarB1              NGPLVVLDNVKFAQFAEIVHFTLPDGTIRSGQVLEVVGTKAIIQVFEGTSGIDAQRTTCE
```

```
DrosophilaB       FTGDILRTPVSEDMLGRVFNGSGKPIDKGPPILAEDFLDIQGQPINPWSRIYPEEMIQTG
HemichordateB     FTGDILRTPVSEDMLGRVFNGSGKPIDKGPPVLAEDFLDIMGQPINPWSRIYPEEMIQTG
AmphioxusBa       FTGDILRTPVSEDMLGRVFNGSGKPIDKGPNILAEDYLDIQGQPINPQSRIYPEEMIQTG
CionaB            FTGDILRIPTSEDMLGRIFNGSGKPIDKGPSVLPEDYLDIQGQPINPESRIYPEEMIQTG
LampreyB          FTGDILRTPVSEDMLGRVFNGSGKPIDKGPPVLAEDYLDIMGQPINPQSRIYPEEMIQTG
AnguillaB1(Ba)    FTGDILRTPVSEDMLGRVFNGSGKPIDRGPMVLAEDYLDIMGQPINPQCRIYPEEMIQTG
ZebrafishBa       FTGDILRTPVSEDMLGRVFNGSGKPIDRGPTVLAEDYLDIMGQPINPQCRIYPEEMIQTG
GarBa             FTGDILRTPVSEDMLGRVFNGSGKPIDRGPSVLAEEYLDIMGQPINPQCRIYPEEMIQTG
MedakaBa          FTGDILRTPVSEDMLGRVFNGSGKPIDRGPNVLAEDYLDIMGQPINPQCRIYPEEMIQTG
SticklebackBa     FTGDILRTPVSEDMLGRVFNGSGKPIDRGPTVLAEDYLDIMGQPINPQCRIYPEEMIQTG
TetraodonBa       FTGDILRTPVSEDMLGRVFNGSGKPIDRGPSVLAEDYLDIMGQPINPQCRIYPEEMIQTG
MouseB2           FTGDILRTPVSEDMLGRVFNGSGKPIDRGPVVLAEDFLDIMGQPINPQCRIYPEEMIQTG
HumanB2           FTGDILRTPVSEDMLGRVFNGSGKPIDRGPVVLAEDFLDIMGQPINPQCRIYPEEMIQTG
CoelacanthB2      ----------------RVFNGSGKPIDRGPTVLAEDFLDIMGQPINPQCRIYPEEMIQTG
XenopusB2         FSGDILRTPVSEDMLGRVFNGSGKPIDRGPSVLAEDYLDIMGQPINPQCRIYPEEMIQTG
GarB2             FTGDILRTPVSEDMLGRVFNGSGKPIDRGPTVMAEDYLDIM-------------------
AnguillaB2        FTGDILRTPVSEDMLGRVFNGSGKPIDRGPMVLAEDYLDIMGQPINPQCRIYPEEMIQTG
ZebrafishB2       FTGDILRTPVSEDMLGRVFNGSGKPIDRGPAVLAEDYLDIMGQPINPQCRIYPEEMIQTG
TetraodonB2       FTGDILRTPVSEDMLGRVFNGSGKPIDRGPAVLAEDFLDIMGQPINPQCRIYPEEMIQTG
MedakaB2          FTGDILRTPVSEDMLGRVFNGSGKPIDRGPAVLAEDYLDIMGQPINPQCRIYPEEMIQTG
MouseB1           FTGDILRTPVSEDMLGRIFNGSGKPIDKGPAVMAEEFLDINGQPINPHDRIYPEEMIQTG
HumanB1           FTGDILRTPVSEDMLGRVFNGSGKPIDKGPVVMAEDFLDINGQPINPHSRIYPEEMIQTG
CoelacanthB1      FTGDILRTPVSEDMLGRVFNGSAKPIDKGPAVMAEDFLDINGQPINPDVRIYPEEMIQTG
XenopusB1         FTGDILRTPVSEDMLGRVFNGSGKPIDKGPTVMAEDYLDINGQPINPYVRTYPEEMIQTG
GarB1             FTGDILRTPVSEDMLGRIFNGSGKSIDKGPAVTAEDFLDINGQPINPQMRIYPEEMIQTG
                                  *:****.*.**:** : .*::***

DrosophilaB       ISAIDVMNSIARGQKIPIFSAAGLPHNEIAAQICRQAGLVKLPGKSVLDDHTDNFAIVFA
HemichordateB     LASIDVMNSIARGQKIPIFSAAGLPHNEIAAQICRQAGLVKHN-KSVMDDHEDNFAIVFA
AmphioxusBa       ISAIDCMNSIARGQKIPIFSAAGLPHNEIAAQICRQGGLVKKQGKSIVDDHDDNFAIVFA
CionaB            ISAIDVMNSIARGQKIPIFSANGLPHNEIAAQICRQGGLVKMPDKSVLDGHDDNFAIVFA
LampreyB          ISAIDTMNSIARGQKIPIFSAAGLPHNEIAAQICRQGGLVKQG-KDTMDYSDDNFAIVFA
AnguillaB1(Ba)    ISAIDGMNSIARGQKIPIFSAAGLPHNEIAAQICRQAGLVQKSKDVMDYS-SENFAIVFA
ZebrafishBa       ISAIDGMNSIARGQKIPIFSAAGLPHNEIAAQICRQAGLVQKSKDVTDYS-SENFAIVFA
GarBa             ISAIDGMNSIARGQKIPIFSAAGLPHNEIAAQICRQAGLVQKSKDVMDYS-ADNFAIVFA
MedakaBa          ISAIDGMNSIARGQKIPIFSAAGLPHNEIAAQICRQAGLVQKSKDVMDYS-AENFAIVFA
SticklebackBa     ISAIDGMNSIARGQKIPIFSAAGLPHNEIAAQICRQAGLVQKSKDVMDYS-SENFAIVFA
TetraodonBa       ISAIDGMNSIARGQKIPIFSAAGLPHNEIAAQICRQAGLVQKSKDVMDYS-ADNFAIVFA
MouseB2           ISAIDGMNSIARGQKIPIFSAAGLPHNEIAAQICRQAGLVKKSKDVVDYS-EENFAIVFA
HumanB2           ISAIDGMNSIARGQKIPIFSAAGLPHNEIAAQICRQAGLVKKSKDVVDYS-EENFAIVFA
CoelacanthB2      ISAIDGMNSIARGQKIPIFSAAGLPHNEIAAQICRQAGLVKKSKDVVDYT-EENFAIVFA
XenopusB2         ISAIDGMNSIARGQKIPIFSAAGLPHNEIAAQICRQAGLVKKSKDVMDYS-EDNFAIVFA
GarB2             ------------------------------------------DVMDYS-EENFAIVFA
AnguillaB2        ISAIDGMNSIARGQKIPIFSAAGLPHNEIAAQICRQAGLVKKSKDVMDYS-ADNFAIVFA
ZebrafishB2       ISAIDGMNSIARGQKIPIFSAAGLPHNEIAAQICRQAGLVKKSKDVMDYS-EDNFAIVFA
TetraodonB2       ISAIDGMNSIARGQKIPIFSAAGLPHNEIAAQICRQAGLVRKSKDVVDYS-EDNFAIVFA
MedakaB2          ISAIDGMNSIARGQKIPIFSAAGLXHNEIAAQICRQAGLVKKSKDVMVYS-EDNFAIVFA
MouseB1           ISPIDVMNSIARGQKIPIFSAAGLPHNEIAAQICRQAGLVKKSKAVLDYH-EDNFAIVFA
HumanB1           ISPIDVMNSIARGQKIPIFSAAGLPHNEIAAQICRQAGLVKKSKAVLDYH-DDNFAIVFA
CoelacanthB1      ISPIDVMNSIARGQKIPIFSAAGLPHNEIAAQICRQAGLVKQSKSVMDFH-DDNFAIVFA
XenopusB1         ISPIDVMNSIARGQKIPIFSAAGLPHNEIAAQICRQAGLVKKSKDVMDYS-DDNFAIVFA
GarB1             LSPIDAMNSIARGQKIPIFSAAGLPHNEIAAQICRQAGLVQQHKSVMDHH-EDNFAIVFA
                                                                     :*******

DrosophilaB       AMGVNMETARFFKQDFEENGSMENVCLFLNLANDP-TIERIITPRLALTAAEFLAYQCEK
HemichordateB     AMGVNMETARFFKQDFEENGSMENVCLFLNLANDP-TIERIITPRLALTCAEFLAYQCEK
AmphioxusBa       AMGVNMETARFFKMDFEENGSMENVCLFLNLANDP-TIERIITPRLALTTAEFLAYQCEK
CionaB            AMGVNMEAARFFKSDFEQHGSMDNVCLFLNLANDYTMIERIITPRIALTTAEFLAYQCEK
LampreyB          AMGVNMETARFFKSDFEENGSMDNVCLFLNLANDP-TIERIITPRLALTTAEFLAYQCEK
AnguillaB1(Ba)    AMGVNMETARFFKSDFEENGSMDNVCLFLNLANDP-TIERIITPRLALTTAEYLAYQCEK
ZebrafishBa       AMGVNMETARFFKSDFEENGSMDNVCLFLNLANDP-TIERIITPRLALTTAEYLAYQCEK
GarBa             AMGVNMETARFFKSDFEENGSMDNVCLFLNLANDP-TIERIITPRLALTTAEYLAYQCEK
MedakaBa          AMGVNMETARFFKSDFEENGSMDNVCLFLNLANDP-TIERIITPRLALTTAEYLAYQCEK
SticklebackBa     AMGVNMETARFFKSDFEENGSMDNVCLFLNLANDP-TIERIITPRLALTSAEYLAYQCEK
TetraodonBa       AMGVNMETARFFKSDFEENGSMDNVCLFLNLANDP-TIERIITPRLALTSAEYLAYQCEK
```

```
MouseB2           AMGVNMETARFFKSDFEENGSMDNVCLFLNLANDP-TIERIITPRLALTTAEFLAYQCEK
HumanB2           AMGVNMETARFFKSDFEENGSMDNVCLFLNLANDP-TIERIITPRLALTTAEFLAYQCEK
CoelacanthB2      AMGVNMETARFFKSDFEENGSMDNVCLFLNLANDP-TIERIITPRLALTTAEFLAYQCEK
XenopusB2         AMGVNMETARFFKSDFEENGSMDNVCLFLNLANDP-TIERIITPRLALTSAEFLAYQCEK
GarB2             AMGVNMETARFFKSDFEENGSMDNVCLFLNLANDP-TIERIITPRLALTTAEFLAYQCEK
AnguillaB2        AMGVNMETARFFKSDFEENGSMDNVCLFLNLANDP-TIERIITPRLALTTAEFLAYQCEK
ZebrafishB2       AMGVNMETARFFKSDFEENGSMDNVCLFLNLANDP-TIERIITPRLALTSAEFLAYQCEK
TetraodonB2       AMGVNMETARFFKSDFEENGSMDNVCLFLNLANDP-TIERIITPRLALTSAEYLAYQCEK
MedakaB2          AMGVNMETARFFKSDFEENGSMDNVCLFLNLANDP-TIERIITPRLALTAAEYLAYQCEK
MouseB1           AMGVNMETARFFKSDFEQNGTMGNVCLFLNLANDP-TIERIITPRLALTTAEFLAYQCEK
HumanB1           AMGVNMETARFFKSDFEQNGTMGNVCLFLNLANDP-TIERIITPRLALTTAEFLAYQCEK
CoelacanthB1      AMGVNMETARFFKSDFEENGSMDNVCLFLNLANDP-TIERIITPRLALTTAEFLAYQCEK
XenopusB1         AMGVNMETARFFKSDFEQNGTMDNVCLFLNLANDP-TIERIITPRLALTEAEYLAYQCEK
GarB1             AMGVNMETARFFKSDFEENGSMDNVCLFLNLANDP-TIERIITPRLALTTAEFLAYQCEK
                  *******:***** ***::*:* ********.**   ********:*** **:*******

DrosophilaB       HVLVILTDMSSYAEALREVSAAREEVPGRRGFPGYMYTDLATIYERAGRVEGRNGSITQI
HemichordateB     HVLVILTDMSSYAEALREVSAAREEVPGRRGFPGYMYTDLATIYERAGRVEGRNGSITQI
AmphioxusBa       HVLVILTDMSSYAEALREVSAAREEVPGRRGFPGYMYTDLATIYERAGRVDGRNGSITQI
CionaB            HVLVILTDMSSYAEALREVSAAREEVPGRRGFPGYMYTDLATIYERAGRVSGRNGSITQI
LampreyB          HVLVILTDMSSYAEALREVSAAREEVPGRRGFPGYMYTDLATIYERAGRVEGRNGSITQI
AnguillaB1(Ba)    HVLVILTDMSSYAEALREVSAAREEVPGRRGFPGYMYTDLATIYERAGRVEGRNGSITQI
ZebrafishBa       HVLVILTDMSSYAEALREVSAAREEVPGRRGFPGYMYTDLATIYERAGRVEGRNGSITQI
GarBa             HVLVILTDMSSYAEALREVSAAREEVPGRRGFPGYMYTDLATIYERAGRIEGRNGSITQI
MedakaBa          HVLVILTDMSSYAEALREVSAAREEVPGRRGFPGYMYTDLATIYERAGRVEGRNGSITQI
SticklebackBa     HVLVILTDMSSYAEALREVSAAREEVPGRRGFPGYMYTDLATIYERAGRVEGRNGSITQI
TetraodonBa       HVLVILTDMSSYAEALREVSAAREEVPGRRGFPGYMYTDLATIYERAGRVEGRNGSITQI
MouseB2           HVLVILTDMSSYAEALREVSAAREEVPGRRGFPGYMYTDLATIYERAGRVEGRNGSITQI
HumanB2           HVLVILTDMSSYAEALREVSAAREEVPGRRGFPGYMYTDLATIYERAGRVEGRNGSITQI
CoelacanthB2      HVLVILTDMSSYAEALREVSAAREEVPGRRGFPGYMYTDLATIYERAGRVEGRNGSITQI
XenopusB2         HVLVILTDMSSYAEALREVSAAREEVPGRRGFPGYMYTDLATIYERAGRVEGRNGSITQI
GarB2             HVLVILTDMSSYAEALREVSAAREEVPGRRGFPGYMYTDLATIYERAGRVEGRNGSITQI
AnguillaB2        HVLVILTDMSSYAEALREVSAAREEVPGRRGFPGYMYTDLATIYERAGRVEGRNGSITQI
ZebrafishB2       HVLVILTDMSSYAEALREVSAAREEVPGRRGFPGYMYTDLATIYERAGRVEGRNGSITQI
TetraodonB2       HVLVILTDMSSYAEALREVSAAREEVPGRRGFPGYMYTDLATIYERAGRVEGRNGSITQI
MedakaB2          HVLVILTDMSSYAEALREVSAAREEVPGRRGFPGYMYTDLATIYERAGRVEGRSGSITQI
MouseB1           HVLVILTDMSSYAEALREVSAAREEVPGRRGFPGYMYTDLATIYERAGRVEGRGGSITQI
HumanB1           HVLVILTDMSSYAEALREVSAAREEVPGRRGFPGYMYTDLATIYERAGRVEGRGGSITQI
CoelacanthB1      HVLVLLTDMSSYAEALREVSAAREEVPGRRGFPGYMYTDLATIYERAGRVEGRNGSITQI
XenopusB1         HVLVILTDMSSYAEALREVSAAREEVPGRRGFPGYMYTDLATIYERAGRVEGRSGSITQI
GarB1             HVLVILTDMSSYAEALREVSAAREEVPGRRGFPGYMYTDLATIYERAGRVDGRQGSITQI
                  ****:********************************************:.** ******

DrosophilaB       PILTMPNDDITHPIPDLTGYITEGQIYVDRQLHNRQIYPPVNVLPSLSRLMKSAIGEGMT
HemichordateB     PILTMPNDDITHPIPDLTGYITEGQIYVDRQLHNRQIYPPINVLPSLSRLMKSAIGEGMT
AmphioxusBa       PILTMPNDDITHPIPDLTGYITEGQIYVERQLHNRQIYPPINVLPSLSRLMKSAIGEGMT
CionaB            PILTMPNDDITHPIPDLTGYITEGQIYIDRQLHNRQIYPPINVLPSLSRLMKSAIGEGMT
LampreyB          PILTMPNDDITHPIPDLTGYITEGQIYVDRQLHNRQIYPPINVLPSLSRLMKSAIGEGMT
AnguillaB1(Ba)    PILTMPNDDITHPIPDLTGYITEGQVYVDRQLHNRQIYPPINVLPSLSRLMKSAIGEGMT
ZebrafishBa       PILTMPNDDITHPIPDLTGYITEGQVYVDRQLHNRQIYPPINVLPSLSRLMKSAIGEGMT
GarBa             PILTMPNDDITHPIPDLTGYITEGQVYVDRQLHNRQIYPPINVLPSLSRLMKSAIGEGMT
MedakaBa          PILTMPNDDITHPIPDLTGYITEGQVYVDRQLHNRQIYPPINVLPSLSRLMKSAIGEGMT
SticklebackBa     PILTMPNDDITHPIPDLTGYITEGQVYVDRQLHNRQIYPPINVLPSLSRLMKSAIGEGMT
TetraodonBa       PILTMPNDDITHPIPDLTGYITEGQVYVDRQLHNRQIYPPINVLPSLSRLMKSAIGEGMT
MouseB2           PILTMPNDDITHPIPDLTGYITEGQIYVDRQLHNRQIYPPINVLPSLSRLMKSAIGEGMT
HumanB2           PILTMPNDDITHPIPDLTGYITEGQIYVDRQLHNRQIYPPINVLPSLSRLMKSAIGEGMT
CoelacanthB2      PILTMPNDDITHPIPDLTGYITEGQIYVDRQLHNRQIYPPINVLPSLSRLMKSAIGEGMT
XenopusB2         PILTMPNDDITHPIPDLTGYITEGQIYVDRQLHNRQIYPPINVLPSLSRLMKSAIGEGMT
GarB2             PILTMPNDDITHPIPDLTGYITEGQIYVDRQLHNRQIYPPINVLPSLSRLMKSAIGEGMT
AnguillaB2        PILTMPNDDITHPIPDLTGYITEGQIYVERQLHNRQIYPPINVLPSLSRLMKSAIGEGMT
ZebrafishB2       PILTMPNDDITHPIPDLTGYITEGQIYVDRQLHNRQIYPPINVLPSLSRLMKSAIGEGMT
TetraodonB2       PILTMPNDDITHPIPDLTGYITEGQIYVDRQLHNRQIYPPINVLPSLSRLMKSAIGEGMT
MedakaB2          PILTMPNDDITHPIPDLTGYITEGQIYVDRQLHNRQIYPPINVLPSLSRLMKSAIGEGMT
MouseB1           PILTMPNDDITHPIPDLTGFITEGQIYVDRQLHNRQVYPPINVLPSLSRLMKSAIGEGMT
HumanB1           PILTMPNDDITHPIPDLTGFITEGQIYVDRQLHNRQIYPPINVLPSLSRLMKSAIGEGMT
```

```
CoelacanthB1        PILTMPNDDITHPIPDLTGFITEGQVYVDRQLHNRQIYPPINVLPSLSRLMKSAIGEEMT
XenopusB1           PILTMPNDDITHPIPDLTGFITEGQIYVDRQLHNRQIYPPINVLPSLSRLMKSAIGEGMT
GarB1               PILTMPNDDITHPIPDLTGFITEGQVYIDRQLHNRQIYPPINVLPSLSRLMKSAIGEGMT
                    ***********:*******:*****:*::*******:***:**************** **

DrosophilaB         RKDHSDVSNQLYACYAIGKDVQAMKAVVGEEALTPDDLLYLEFLTKFEKNFISQGNYENR
HemichordateB       RKDHADVSNQLYANYAIGKDVQAMKAVVGEEALTPDDLLYLEFLGKFEKNFIAQGAYENR
AmphioxusBa         RKDHADVSNQLYACYAIGKDVQAMKAVVGEEALTPDDLLYLEFLSKFEKNFINQGPYDNR
CionaB              RKDHSDVSNQLYANYAIGKDVQAMKAVVGEEALTQDDMLYLEFLTKFEKSFIAQGSYENR
LampreyB            RKDHSDVSNQLYACYAIGKDVQAMKAVVGEEALTPDDLLYLEFLQKFEKTFIAQGAYENR
AnguillaB1(Ba)      RKDHADVSNQLYACYAIGKDVQAMKAVVGEEALTSDDLLYLEFLQKFEKNFIAQGPYDNR
ZebrafishBa         RKDHADVSNQLYACYAIGKDVQAMKAVVGEEALTSDDLLYLEFLQKFEKNFIAQGAYDNR
GarBa               RKDHADVSNQLYACYAIGKDVQAMKAVVGEEALTSDDLLYLEFLQKFEKNFIAQGPYDNR
MedakaBa            RKDHADVSNQLYACYAIGKDVQAMKAVVGEEALTSDDLLYLEFLQKFEKNFIAQGPYDNR
SticklebackBa       RKDHADVSNQLYACYAIGKDVQAMKAVVGEEALTSDDLLYLEFLQKFEKNFIAQGPYDNR
TetraodonBa         RKDHADVSNQLYACYAIGKDVQAMKAVVGEEALTSDDLLYLEFLQKFEKNFISQGPYDNR
MouseB2             RKDHADVSNQLYACYAIGKDVQAMKAVVGEEALTSDDLLYLEFLQKFEKNFITQGPYENR
HumanB2             RKDHADVSNQLYACYAIGKDVQAMKAVVGEEALTSDDLLYLEFLQKFERNFIAQGPYENR
CoelacanthB2        RKDHADVSNQLYACYAIGKDVQAMKAVVGEEALTSDDLLFLEFLQKFERNFISQGPYENR
XenopusB2           RKDHSDVSNQLYACYAIGKDVQAMKAVVGEEALTSDDLLYLEFLHKFEKNFIAQGPYDNR
GarB2               RKDHADVSNQLYACYAIGKDVQAMRAVVGEEALTADDLLYLEFLQKFEKNFISQGAYENR
AnguillaB2          RKDHADVSNQLYACYAIGKDVQAMKAVVGEEALTSDDLLYLEFLQKFEKNFIAQGAYENR
ZebrafishB2         RKDHSDVSNQLYACYAIGKDVQAMKAVVGEEALTSDDLLYLEFLQKFERNFISQGAYENR
TetraodonB2         RRDHSDVSNQLYACYAIGKDVQAMKAVVGEEALTPDDLLYLEFLTKFEKNFISQGAYENR
MedakaB2            RKDHADVSNQLYACYAIGKDVQAMKAVVGEEALTADDLLYLEFLSKFEKNFISQGAYENR
MouseB1             RKDHGDVSNQLYACYAIGKDVQAMKAVVGEEALTSEDLLYLEFLQKFEKNFITQGPYENR
HumanB1             RKDHGDVSNQLYACYAIGKDVQAMKAVVGEEALTSEDLLYLEFLQKFEKNFINQGPYENR
CoelacanthB1        RKDHADVSNQLYACYAIGKDVQAMKAVVGEEALTSDDLLYLEFLQKFEKQFINQGPYENR
XenopusB1           RKDHGDVSNQLYACYAIGKDVQAMKAVVGEEALSSDDLLYLEFLQKFEKQFIAQGPYENR
GarB1               RNDHADVSNQLYACYAIGKDVQAMKAVVGEEALSQEDLLYLEFLHKFERRFISQGPYENR
                    *.**.******** **********:********: :*:*:**** ***: ** ** *:**


DrosophilaB         TVFESLDIGWQLLRIFPKEMLKRIPASILAEFYPRDSRH---------------------
HemichordateB       SVFDSLDIGWQLLRIFPKEMLKRIPQSTLSEFYPRDSTRGGH------------------
AmphioxusBa         SIYESLDIGWSLLRIFPKQMLKRIPEKTLNEFYPRESRTTTK------------------
CionaB              TVFDSLDIGWELLRIFPKEMLKRIPRNVINEYYPRKKPAVPTK-----------------
LampreyB            TIFESLDIGWQLLRIFPKEMLKRIPQSTIAEFYPREANAAKL------------------
AnguillaB1(Ba)      TVYETLDIGWQLLRIFPKEMLKRIPQSTLAEFYPRESAARH-------------------
ZebrafishBa         TVFETLDIGWQLLRIFPKEMLKRIPQSTLAEFYPRESAARHGAS----------------
GarBa               TVFETLDIGWQLLRIFPKEMLKRIPQSTLAEFYPRESAARH-------------------
MedakaBa            TVYETLDIGWQLLRIFPKEMLKRIPQSTLAEFYPRESAARH-------------------
SticklebackBa       TVYETLDIGWQLLRIFPKEMLKRIPQSTLAEFYPRESAARH-------------------
TetraodonBa         TVYETLDIGWQLLRIFPKEMLKRIPQSTLSEFYPRESSSRH-------------------
MouseB2             TVYETLDIGWQLLRIFPKEMLKRIPQSTLSEFYPRDSAKH--------------------
HumanB2             TVFETLDIGWQLLRIFPKEMLKRIPQSTLSEFYPRDSAKH--------------------
CoelacanthB2        TVYETLDIGWQLLRIFPKEMLKRIPQSILAEFYPRDSAAKH-------------------
XenopusB2           TVYETLDIGWQLLRIFPKELLKRIPQSTLAEFYPRDSSAKH-------------------
GarB2               SVYETLDIGWQLMRIFPKEMLKRIPQSTLAEFYPRDSKH---------------------
AnguillaB2          TVFETLDIGWQLLRIFPKEMLKRIPQSTLAEFYPRDSKH---------------------
ZebrafishB2         TVFETLDIGWQLLRIFPKEMLKRIPQSTLAEFYPRDSKH---------------------
TetraodonB2         SVFETLDIGWQLMRIFPKEMLKRIPQATLAEFYPREAKH---------------------
MedakaB2            SVFETLDVGWQLMRIFPKEMLKRIPQSTLAQFYPREAKH---------------------
MouseB1             TVFESLDLGWKLLRIFPKEMLKRIPQSMTDEFYSRQGAQQDPASDTAL------------
HumanB1             SVFESLDLGWKLLRIFPKEMLKRIPQAVIDEFYSREGALQDLAPDTAL------------
CoelacanthB1        TVFESLDIGWQLLRIFPKEMLKRIPESVLSEFYPREARANTQQALFTIL-----------
XenopusB1           SIFESLDIGWQLLRIFPKELLKRIPESMLAEFYPRESRAHGEGPSKTR------------
GarB1               SVFESLDIGWELLRLFPKEMLKRIPVSILNEFYSRDGPRQPPNKPQRQPTPSAPETESPP
                    :::::**:**.*:*:***::*****     ::*.*.
```

A

```
Gar Exon7             agATTGCTGCACAGATATGTCGTCAGGCTGGCCTTGTTCAGAAATCCAAAGATGTGATGG
Coelacanth ΨExon7     agACTGTTGCCCAGCTCAACCATCAAGCTGGCCTGTTACAAAAATCCAAAGATGTCATGG

Gar Exon7             ATTATAGTGCGGACAACTTTGCCATCGTGTTTGCGGCTATGGGAgt
Coelacanth ΨExon7     ACTTCAGTGCTAAGAATTTTACTATTGTGTTCACAGCAGTTGATtt


Gar Exon7             IAAQICRQAGLVQKSKDVMDYSADNFAIVFAAMG
Coelacanth ΨExon7     TVAQLNHQAGLLQKSKDVMDFSAKNFTIVFTAVD
```

B

```
Gar Exon12            agATATACCCTCCTATCAATGTGTTGCCTTCTCTGTCTCGACTGATGAAATCAGCTATTG
Human ΨExon12         agGTTTGCCCTCCAATCAGTGGTCTGCCTTCATTTTCCAGATTGATGAAGTCTGCCTCAG
Turtle ΨExon12        agATTTATCCTCCAATCAGTGTTCCATCTTCATTGTCCAGGCAGATGAAGCATGCCATAG

Gar Exon12            GAGAAGGG-ATGACACGCAAAGACCACGCTGATGTCTCCAACCAACTGgt
Human ΨExon12         -AGAAGGG-GGTAGGTAACAAGACCGTGCCGATGTGTCTAATCAGCTGgg
Turtle ΨExon12        TAGAAGGGAATGACTAGGAAATATCATGCCAAGGTCTCTAATCAAATGgt


Gar Exon12            IYPPINVLPSLSRLMKSAIGEG-MTRKDHADVSNQL
Human ΨExon12         VCPPISGLPSFSRLMKSASXEG-GR*QDRADVSNQL
Turtle ΨExon12        IYPPISVPSSLSRQMKHAIVEGXMTRKYHAKVSNQM
```

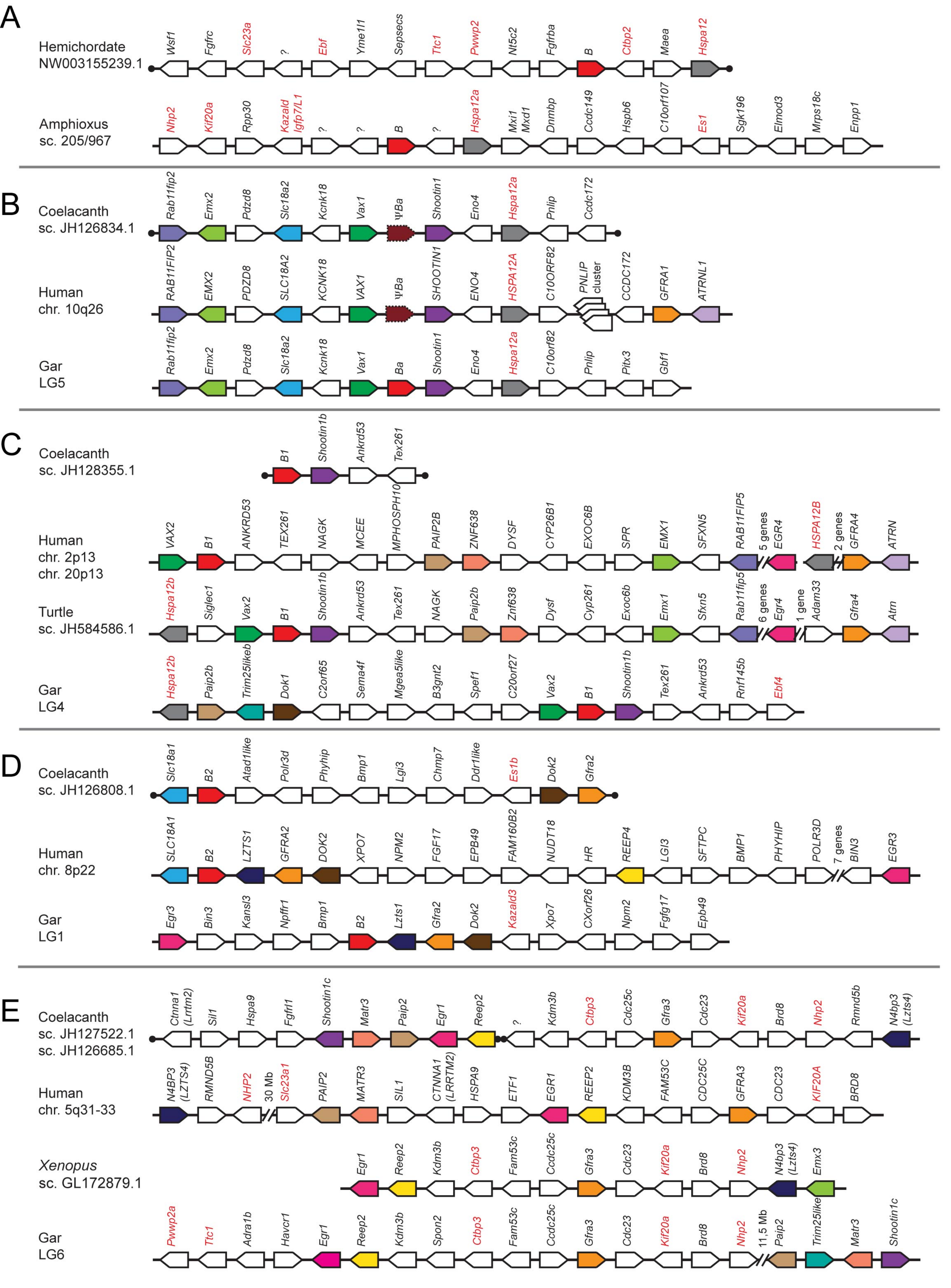
A
Hemichordate
NW003155239.1
Wsf1
Fgfrc
Slc23a
?
Ebf
Yme1l1
Sepsecs
Ttc1
Pwwp2
Nt5c2
Fgfrba
B
Ctbp2
Maea
Hspa12
Amphioxus
sc. 205/967
Nhp2
Kif20a
Rpp30
Kazald Igfp7/L1
?
?
B
?
Hspa12a
Mxi1 Mxd1
Dnmbp
Ccdc149
Hspb6
C10orf107
Es1
Sgk196
Elmod3
Mrps18c
Enpp1
B
Coelacanth
sc. JH126834.1
Rab11fip2
Emx2
Pdzd8
Slc18a2
Kcnk18
Vax1
ΨBa
Shootin1
Eno4
Hspa12a
Pnlip
Ccdc172
Human
chr. 10q26
RAB11FIP2
EMX2
PDZD8
SLC18A2
KCNK18
VAX1
ΨBa
SHOOTIN1
ENO4
HSPA12A
C10ORF82
PNLIP cluster
CCDC172
GFRA1
ATRNL1
Gar
LG5
Rab11fip2
Emx2
Pdzd8
Slc18a2
Kcnk18
Vax1
Ba
Shootin1
Eno4
Hspa12a
C10orf82
Pnlip
Pitx3
Gbf1
C
Coelacanth
sc. JH128355.1
B1
Shootin1b
Ankrd53
Tex261
Human
chr. 2p13
chr. 20p13
VAX2
B1
ANKRD53
TEX261
NAGK
MCEE
MPHOSPH10
PAIP2B
ZNF638
DYSF
CYP26B1
EXOC6B
SPR
EMX1
SFXN5
RAB11FIP5
5 genes
EGR4
HSPA12B
2 genes
GFRA4
ATRN
Turtle
sc. JH584586.1
Hspa12b
Siglec1
Vax2
B1
Shootin1b
Ankrd53
Tex261
NAGK
Paip2b
Znf638
Dysf
Cyp261
Exoc6b
Emx1
Sfxn5
Rab11fip5
6 genes
Egr4
1 gene
Adam33
Gfra4
Atrn
Gar
LG4
Hspa12b
Paip2b
Trim25likeb
Dok1
C2orf65
Sema4f
Mgea5like
B3gnt2
Spef1
C20orf27
Vax2
B1
Shootin1b
Tex261
Ankrd53
Rnf145b
Ebf4
D
Coelacanth
sc. JH126808.1
Slc18a1
B2
Atad1like
Polr3d
Phyhip
Bmp1
Lgi3
Chmp7
Ddr1like
Es1b
Dok2
Gfra2
Human
chr. 8p22
SLC18A1
B2
LZTS1
GFRA2
DOK2
XPO7
NPM2
FGF17
EPB49
FAM160B2
NUDT18
HR
REEP4
LGI3
SFTPC
BMP1
PHYHIP
POLR3D
7 genes
BIN3
EGR3
Gar
LG1
Egr3
Bin3
Kansl3
Npffr1
Bmp1
B2
Lzts1
Gfra2
Dok2
Kazald3
Xpo7
CXorf26
Npm2
Fgfg17
Epb49
E
Coelacanth
sc. JH127522.1
sc. JH126685.1
Ctnna1 (Lrrtm2)
Sil1
Hspa9
Fgfrl1
Shootin1c
Matr3
Paip2
Egr1
Reep2
?
Kdm3b
Ctbp3
Cdc25c
Gfra3
Cdc23
Kif20a
Brd8
Nhp2
Rmnd5b
N4bp3 (Lzts4)
Human
chr. 5q31-33
N4BP3 (LZTS4)
RMND5B
NHP2
30 Mb
Slc23a1
PAIP2
MATR3
SIL1
CTNNA1 (LRRTM2)
HSPA9
ETF1
EGR1
REEP2
KDM3B
FAM53C
CDC25C
GFRA3
CDC23
KIF20A
BRD8
Xenopus
sc. GL172879.1
Egr1
Reep2
Kdm3b
Ctbp3
Fam53c
Ccdc25c
Gfra3
Cdc23
Kif20a
Brd8
Nhp2
N4bp3 (Lzts4)
Emx3
Gar
LG6
Pwwp2a
Ttc1
Adra1b
Havcr1
Egr1
Reep2
Kdm3b
Spon2
Ctbp3
Fam53c
Ccdc25c
Gfra3
Cdc23
Kif20a
Brd8
Nhp2
11,5 Mb
Paip2
Trim25like
Matr3
Shootin1c

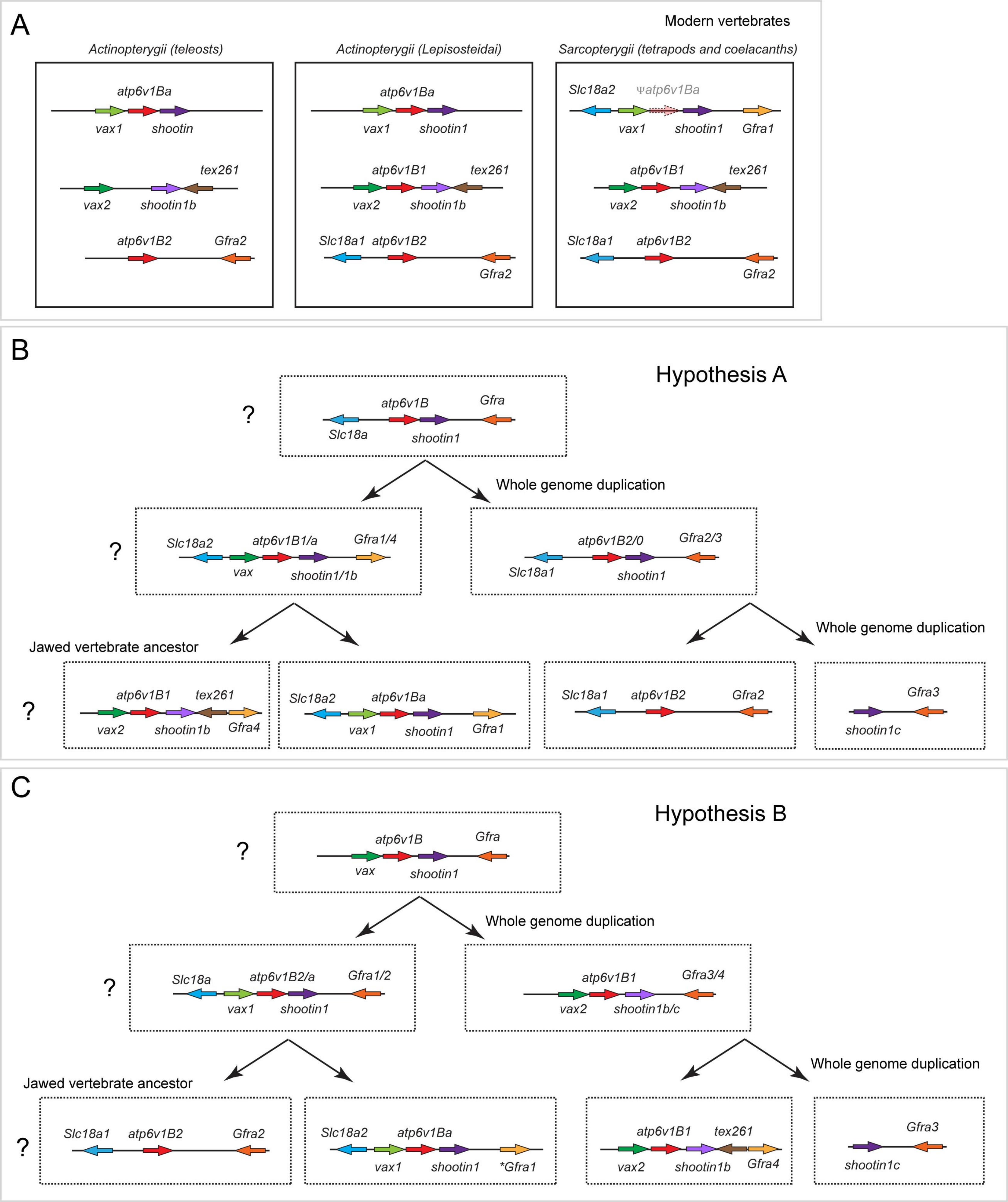
A
Modern vertebrates
Actinopterygii (teleosts)
atp6v1Ba
vax1
shootin
tex261
vax2
shootin1b
atp6v1B2
Gfra2
Actinopterygii (Lepisosteidai)
atp6v1Ba
vax1
shootin1
atp6v1B1
tex261
vax2
shootin1b
Slc18a1
atp6v1B2
Gfra2
Sarcopterygii (tetrapods and coelacanths)
Slc18a2
Ψatp6v1Ba
vax1
shootin1
Gfra1
atp6v1B1
tex261
vax2
shootin1b
Slc18a1
atp6v1B2
Gfra2
B
Hypothesis A
?
atp6v1B
Gfra
Slc18a
shootin1
Whole genome duplication
?
Slc18a2
atp6v1B1/a
Gfra1/4
vax
shootin1/1b
atp6v1B2/0
Gfra2/3
Slc18a1
shootin1
Whole genome duplication
Jawed vertebrate ancestor
?
atp6v1B1
tex261
vax2
shootin1b
Gfra4
Slc18a2
atp6v1Ba
vax1
shootin1
Gfra1
Slc18a1
atp6v1B2
Gfra2
Gfra3
shootin1c
C
Hypothesis B
?
atp6v1B
Gfra
vax
shootin1
Whole genome duplication
?
Slc18a
atp6v1B2/a
Gfra1/2
vax1
shootin1
atp6v1B1
Gfra3/4
vax2
shootin1b/c
Whole genome duplication
Jawed vertebrate ancestor
?
Slc18a1
atp6v1B2
Gfra2
Slc18a2
atp6v1Ba
vax1
shootin1
*Gfra1
atp6v1B1
tex261
vax2
shootin1b
Gfra4
Gfra3
shootin1c
